# Identification of Genes Interacting with *rnt-1* Through Large-Scale RNAi Screening in *Caenorhabditis elegans*

**DOI:** 10.1534/g3.113.007898

**Published:** 2013-10-01

**Authors:** Kiho Lee, Jiwon Shim, Jihyun Lee, Junho Lee

**Affiliations:** * Department of Biological Sciences, Seoul National University, Seoul, South Korea 151-742; ^†^Department of Biophysics and Chemical Biology, Seoul National University, Seoul, South Korea 151-742; ^‡^Department of Medicine, Infectious Diseases Division, Rhode Island Hospital, Warren Alpert Medical School of Brown University, Providence, Rhode Island 02903; ^§^Department of Molecular, Cell and Developmental Biology, University of California, Los Angeles, California 90095

**Keywords:** RNAi, RUNX, CDK8, mediator, genetic interaction

## Abstract

Although many critical roles of the RUNX family proteins have already been identified, little attention has been given to how these proteins interact with other factors. Elucidating RUNX protein interactions will help extend our understanding of their roles in normal development and tumorigenesis. In this study, we performed large-scale RNAi screening to identify genes that genetically interact with *rnt-1*, the sole homolog of RUNX protein in the nematode *Caenorhabditis elegans*. To this end, we took advantage of the fact that *C. elegans* can survive a severe loss of RNT-1 function with only mild phenotypes, and we looked for genes that caused a synthetic phenotype in the *rnt-1* mutant background. We identified seven genes, three of which (*cdk-8*, *cic-1*, and *sur-2*) are involved in transcription, two of which (*pgp-2* and *cct-5*) are involved in stress response, and two of which (D2045.7 and W09D10.4) are involved in signaling cascades, according to their functional gene ontology terms. We further confirmed that the CDK8-containing mediator complex genetically interacts with RNT-1 by showing that knockdown of each component of the CDK8 mediator complex caused a synthetic phenotype, that is, the exploded intestine through the vulva (Eiv) phenotype, in the *rnt-1* mutant background. We also identified a putative target gene, *acs-4*, which is regulated by the RNT-1 and CDK8 mediator complex. Our results strengthen the notion that the CDK8 mediator complex may also act together with RUNX proteins in mammals.

The RUNX genes encode a family of transcription factors that contain a conserved DNA-binding Runt domain. Roles of RUNX genes in various aspects of animal development, such as the development of hematopoietic stem cells, bone, and neuronal cells, have been genetically elucidated through extensive studies of mutations in RUNX genes (Blyth *et al.* 2005). For instance, translocations on RUNX1 loci were found to occur most commonly in acute leukemia (Osato *et al.* 1999; Michaud *et al.* 2002; Osato 2004). Mutations of RUNX1 in mice also result in abnormal development of cells in nervous system, endothelial cells, and immune cells (Wang *et al.* 1996). Mutations of RUNX2 in humans induce incomplete development of osteoblasts, which results in cleidocranial dysplasia and osteosarcoma (Banerjee *et al.* 1997; Werner *et al.* 1999). Further studies of mutations in mice identified that RUNX2 is critical for maturation of osteoblasts in osteogenesis (Otto *et al.* 1997; Aberg *et al.* 2004). Elegant studies in mice revealed that RUNX3 is expressed in multiple tissues, such as endothelial cells in the gastrointestinal tract, T cells, dendritic cells, and neuronal cells, and that RUNX3 has roles in both cell proliferation and differentiation (Bangsow *et al.* 2001; Fukamachi and Ito 2004). Interestingly, mutations in RUNX1 and RUNX2 showed the tendency of haploinsufficiency not only in mice but also in humans. Familial platelet disorder, which is caused by haploinsufficiency of RUNX1, leads to acute myelogenous leukemia (Song *et al.* 1999). Cleidocranial dysplasia, which is induced by mutations in RUNX2, was found to be the result of a haploinsufficient mutation on RUNX2 (Mundlos *et al.* 1997).

It is well-known that a major function of RUNX genes in cells is to regulate the balance between cell proliferation and differentiation (Coffman 2003). Because the decision between cell proliferation and differentiation must reflect the precise cellular environment, the roles and action mechanism of RUNX genes have to be exerted in a context-dependent manner (Braun and Woollard 2009). To reflect precise cellular status, multiple regulations including various splicing forms, overlapped expression of RUNX genes, regulation by upstream activators, numerous RUNX-targeted genes (Cohen 2009), and posttranscriptional regulations (Bae and Lee 2006) were identified for RUNX genes. In addition, RUNX proteins do not act alone, but in conjunction with cofactors; most RUNX proteins form heterodimers with evolutionarily conserved cofactors such as CBFβ. Although rich information on the players that act with RUNX proteins has been obtained in the past, the interacting partners that act transiently, weakly, or indirectly with RUNX proteins have, so far, been overlooked or ignored. Therefore, it would be worth using a genetic model system that allows one to identify genetic interactions of genes by examining the resulting phenotypes.

The nematode *Caenorhabditis elegans* is a good system for studying genetic interaction of RUNX proteins because there is a sole ortholog of RUNX, *rnt-1* (Nam *et al.* 2002; Lee *et al.* 2004), and the existing mutations of *rnt-1* are not lethal. For example, the genetic interaction of *rnt-1* with *cki-1*, a cyclin-dependent kinase inhibitor, allowed identification of the role of *rnt-1* in cell fate commitment in the hypodermal seam cell division, which is similar to the functions of mammalian RUNXs (Nimmo *et al.* 2005). Also, *bro-1*, a cofactor of *rnt-1*, interacts with *rnt-1* and its mutation or knockdown affects hypoplasia of hypodermal seam cells (Kagoshima *et al.* 2007; Shim and Lee 2008). In this study, we screened genes that genetically interact with *rnt-1* through a large-scale RNAi screening in the viable *rnt-1* mutant allele *rnt-1(ok351)*. We identified seven genes that genetically interact with *rnt-1* by observation of the phenotype that is caused by the reduction of both genes’ functions but not by either one alone. We then focused on the components of the CDK8 mediator complex and showed that all of them genetically interact with *rnt-1*. Disruption of both *rnt-1* and a component of the CDK8 mediator complex showed the "exploded intestine through the vulva" phenotype. We demonstrate that *acs-4* is a putative target gene that is regulated simultaneously by *rnt-1* and the CDK8-containing mediator complex.

## Materials and Methods

### Nematode strains

The Bristol N2 strain was used as the wild-type *C. elegans* strain. The *rnt-1(ok351)* mutant strain was obtained from the Caenorhabditis Genetics Center (CGC, Minneapolis, MN), and the *rnt-1(tm491)*, *rnt-1(tm388)*, and *cdk-8(tm1238)* mutant strains were kind gifts from National Bioresources Project (Tokyo, Japan). The *rnt-1cdk-8* double mutants were generated by using standard *C. elegans* techniques and confirmed by single-worm PCR to identify deletions. All strains were grown at 20° on standard Nematode growth media plates (Brenner 1974).

### Feeding RNAi screening

The RNAi library by J. Ahringer (Cambridge, UK), which covers >80% of *C. elegans* open reading frames, was used in the screen. We screened genes on chromosomes I and III. All *Escherichia coli* strains were streaked and cultured with ampicillin on Luria broth. Before worms were fed with *E. coli* strains, 1 mM IPTG was treated to induce ds RNA transcription of the target genes. The phenotypes were observed in the progeny from the mothers that had been subject to RNAi from their L4 stage. We compared phenotypes of wild-type N2 and *rnt-1(ok351)* animals after each RNAi and further confirmed the phenotypes with other alleles, *rnt-1(tm388)* and *rnt-1(tm491)*, at least three times. HT115 bacteria carrying L4440, the plasmid of the empty vector pPD129.36, were used as a control RNAi.

### Microscopy and imaging

To detect RNAi-induced phenotypes, worms were paralyzed with 1 mM levamisole and mounted on 5% agar pads. DIC images were observed using a Zeiss Axioplan 2 microscope.

### Identification of candidate target genes from database

To find genes whose knockdown causes the exploded intestine through the vulva phenotype, we identified candidate target genes from the WormMart at WormBase homepage using "exploded through vulva" as keywords (http://www.wormbase.org). To select genes that harbor the RNT-1 binding sites, R(G/T)ACCRCA, in their promoter regions, we identified the numbers and locations of the RNT-1 binding sites in the 2-kb region of the promoters by visual inspection using the CLC sequence viewer 6 (http://www.clcbio.com).

### RNA preparation, reverse-transcription, and quantitative real-time PCR analysis

Total RNA of mixed-stage worms was isolated with TRIzol reagent (Invitrogen) by freeze–thaw method given in the standard protocol served with the manufacturer’s manual. cDNA was synthesized with RevertAid M-MuLV reverse-transcriptase (Fermentas Canada) using oligo-dT primer and subjected to PCR amplification. Real-time quantitative PCR was performed with BIO-RAD iQ SYBR Green Supermix in iQ5 as described in the manufacturer’s manual. Primers used in quantitative real-time PCR were generated by Primer3 (http://frodo.wi.mit.edu) with default setting. All the results of quantitative real-time PCR were triplicated in each of three independent samples. The *act-1* gene was used for normalization. Student *t* test was used for statistical analyses.

## Results and Discussion

### The rationale of this study

**Figure 1:**
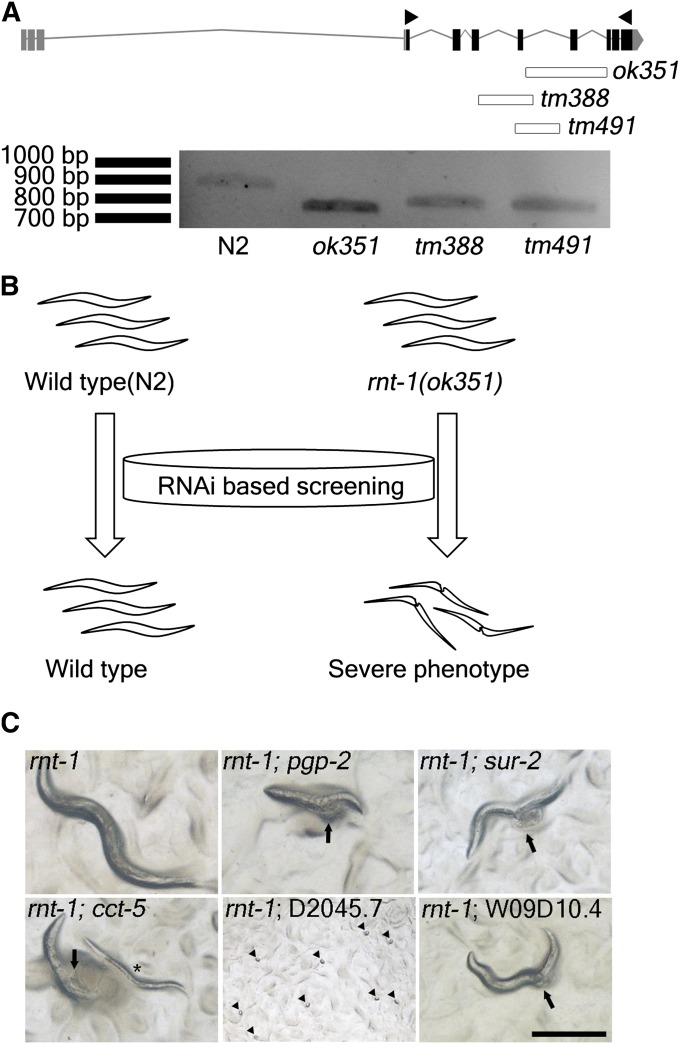
RNAi screening to identify genes that show a synthetic phenotype with *rnt-1*. (A) The gene structure of *rnt-1* and its deletion mutant alleles. The white bars in the diagram indicate the regions deleted by the *rnt-1* mutant alleles. Arrowheads indicate a pair of primers that target full-length of *rnt-1* transcripts. The bottom panel shows the *rnt-1* cDNA products, which were amplified from wild-type N2, *ok351*, *tm388*, and *tm491* strains. (B) The scheme of RNAi screening. We performed unbiased RNAi screening with knockdown of genes on chromosomes I and III. We screened for the genes that showed severe morphological defects in the *rnt-1* mutant background, but not in the wild-type background. (C) Images of synthetic phenotypes observed in *rnt-1* mutant animals after RNAi of each gene listed. Arrows indicate the sites where rupture occurred. Arrowheads indicate dead embryos. *An arrested worm at its larval stage. The scale bar is 200 μm.

Although RUNX genes in mammals such as RUNX1 and RUNX2 are known to be haploinsufficient (Blyth *et al.* 2005), this seems not to be the case for *rnt-1* in *C. elegans* because even homozygous animals that possess any of the three existing in *C. elegans* survive and do not show severe phenotypes. It is possible that *rnt-1* deletion mutant alleles may not be null mutations, although they delete considerable portions of the coding regions. Larger deletions have not been isolated despite extensive random mutagenesis followed by PCR-based screening (J. H. Ahnn, personal communication), and the phenotype caused by *rnt-1* RNAi includes embryonic lethality, which is obviously more severe than that of existing deletion mutations. In addition, there is no chromosomal deficiency available that uncovers the *rnt-1* locus, suggesting that *rnt-1* in *C. elegans* may be haploinsufficient and that the three existing *rnt-1* deletion mutants are not null, but rather reduction-of-function mutations. Consistently, we were able to detect *rnt-1* transcripts RT-PCR even though they were produced as truncated transcripts (Figure 1A, bottom panel). Reduction-of-function mutations are not the best resources for genetics because they make it difficult to determine the exact function of a gene. However, they can sometimes serve as precious tools for elucidating genetic interactions, which otherwise could not be identified because of lethality. We reasoned that the existing deletion mutations of *rnt-1* provide a sensitized genetic background so that one could identify interacting genes by looking for synthetic phenotypes.

### Identification of genes that interact with *rnt-1* by RNAi screening for synthetic phenotypes

Although the *rnt-1(ok351)* allele has the largest deletion within the coding region, it causes the mildest phenotype among the deletion mutants, that is, a slightly short body length and delayed development. We therefore decided to use *ok351* allele as our sensitized genetic background for synthetic phenotype screening. To isolate genes that genetically interact with *rnt-1*, we performed RNAi screening for genes whose RNAi caused an obvious degenerative phenotype only in the *rnt-1(ok351)* background, but not in the wild-type background (Figure 1B). Among approximately 5000 genes on chromosomes I and III that we screened, we found that seven genes caused an *rnt-1*–dependent synthetic phenotype (Figure 1C, summarized in Table 1). The synthetic phenotypes, such as ruptured (Thastrup *et al.* 1990), exploded intestine through vulva (Eiv), larval arrest (Lva), and embryonic lethality (Emb), were observed. RNAi of *pgp-2* and W09D10.4 showed ruptured phenotype. However, *sur-2*, *cdk-8*, and *cic-1* exhibited an Eiv phenotype. Knockdown of *cct-5* caused ruptured phenotype, but Lva also occurred in this case. We also found that RNAi of D2045.7 in the *rnt-1* mutant background induced complete embryonic lethality. Among them, *cdk-8*, *cic-1*, and *sur-2* can be grouped as components of the transcriptional machinery, *pgp-2* and *cct-5* can be grouped as stress-related genes, and D2045.7 and W09D10.4 can be grouped as components of signal cascade, as categorized by the functional GO terms. *cdk-8* and *cic-1* are orthologs of the components of the CDK8 mediator complex, CDK8 and CycC, respectively. *sur-2* is the ortholog of MED23, which is a member of the mediator complex. The mediator complex is a core regulatory integrator that links DNA binding factors and transcriptional machinery (Casamassimi and Napoli 2007). These results suggest that the function of RNT-1 as a transcription factor is closely related with specific transcriptional machinery components in *C. elegans*. *pgp-2* was cloned as an ortholog of multidrug resistance gene (Lincke *et al.* 1992) and is required for the formation of gut granules and lysosome-related fat storage (Schroeder *et al.* 2007). *cct-5* is a component of cytosolic chaperonin (Leroux and Candido 1995). It was reported that knockdown of *cct-5* might increase the expression of *gst-4* through increased oxidative stress evoked by unidentified mechanisms (Kahn *et al.* 2008). The fact that *rnt-1* responds to environmental stress (Lee *et al.* 2012) led us to speculate that a severe defect in the response to stresses may have induced the synthetic phenotype observed. It would be of interest to dissect the molecular aspects of the synthetic phenotypes caused by the reduction of both *cct-5* and *rnt-1* genes. In addition, the mechanism of RNT-1 interaction with signaling components such as D2045.7 and W09D10.4 may shed light on the new action mode of RUNX proteins.

**Table 1:** Genes that were positively selected with severe morphological defects

Sequence Name	Gene Name	LG	Description	Phenotype
C34G6.4	*pgp-2*	I	ABC transporter family, multidrug resistance family	Rup
F39B2.4	*sur-2*	I	Mediator complex component, MED23	Eiv
F39H11.3	*cdk-8*	I	Cyclin-dependent serine/threonine protein kinase, CDK8	Eiv
C07G2.3	*cct-5*	III	Cytosolic chaperonin	Rup, Lva
D2045.7		III	Serine/threonine protein kinase/TGFβ-stimulated factor	Emb
H14E04.5	*cic-1*	III	CDK8 kinase–activating protein, Cyclin C	Eiv
W09D10.4		III	Serine/threonine protein phosphatase	Rup

Rup, ruptured; Eiv, exploded intestine through vulva; Lva, larval arrest; Emb, embryonic lethality.

The *bro-1* gene, located on chromosome I, is a cofactor of RNT-1, and double mutations in *rnt-1* and *bro-1* are known to cause a similar phenotype as the exploded intestine through the vulva (Kagoshima *et al.* 2007). However, because *bro-1* RNAi clone was not included in the Ahringer library, we have not tested the gene in our screening. Likewise, we may have missed additional genes that interact with *rnt-1* because we did not screen genes on chromosomes other than chromosomes I and III. Screening of more chromosomes may result in the identification of new players in RNT-1 action.

### CDK8 mediator complex genetically interacts with *rnt-1*

**Figure 2:**
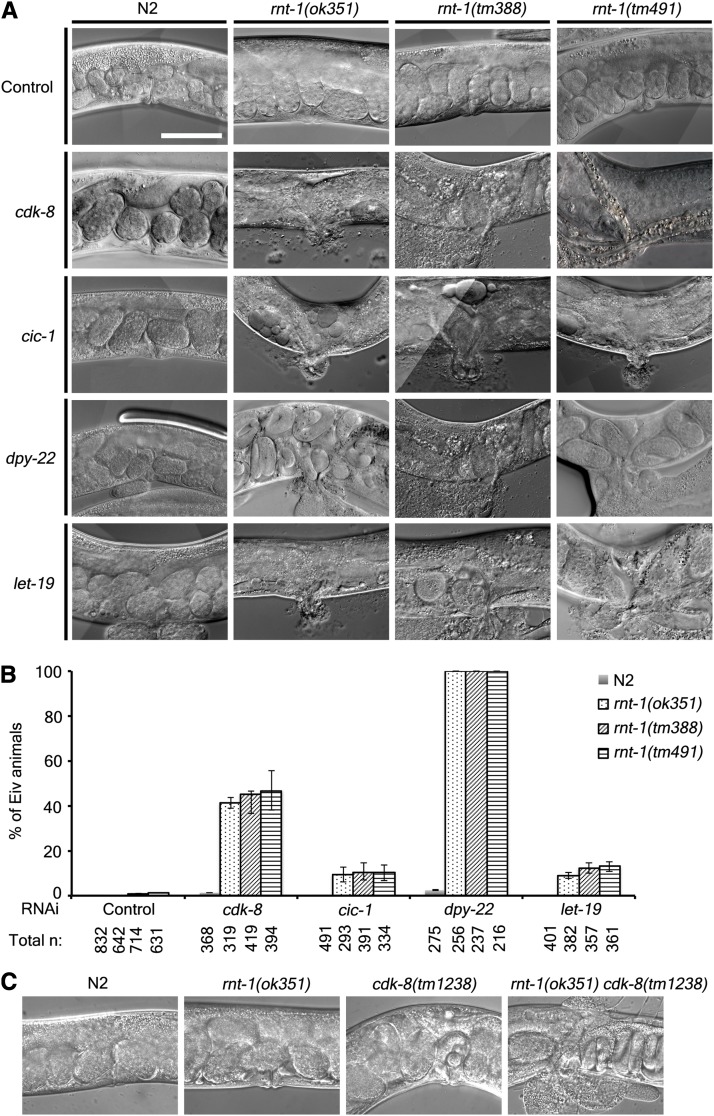
CDK8 mediator complex genetically interacts with *rnt-1*. (A) DIC images of animals with the genotype of wild-type, *rnt-1(ok351)*, *rnt-1(tm388)*, or *rnt-1(tm491)*. Each worm was grown in a control or CDK8 mediator complex RNAi plate, and young adult worms were observed. Note that the intestine, not the gonad, was leaked out through the vulva. (B) The percentage of exploded intestine through vulva (Eiv) animals in wild-type and *rnt-1* mutant animals treated with RNAi of CDK8 mediator complex components. Total n represents total number of worms examined. (C) DIC images of the vulva region of *rnt-1(ok351) cdk-8(tm1238)* double mutants. White bar indicates a scale bar (50 μm).

RNAi of *cdk-8* and *cic-1* in the *rnt-1* mutant background caused a similar phenotype to each other, that is, the Eiv phenotype (Figure 2A). We examined the possibility that other components of the CDK8 mediator complex such as *dpy-22* and *let-19* also genetically interact with *rnt-1*. When we knocked down *dpy-22* or *let-19* in the *rnt-1* mutant background, we obtained the same Eiv phenotype, but not in the wild-type background (Figure 2A). We tested three deletion mutations, *rnt-1(ok351)*, *rnt-1(tm388)*, and *rnt-1(tm491)*, and we observed similar effects (Figure 2A). Interestingly, RNAi of *cdk-8* and *dpy-22* greatly increased the incidence of Eiv phenotype compared to RNAi of other components. To further clarify the genetic interaction between *rnt-1* and *cdk-8*, we generated and examined the *rnt-1(ok351) cdk-8(tm1238)* double mutants. The double mutants were maintained only as heterozygous for *rnt-1* because of the lethality attributable to protruded intestine through the vulva phenotype. We found that 95.4% of the double mutants, which were confirmed by single-worm PCR, exhibited the Eiv phenotype (n = 22; Figure 2C), whereas *rnt-1(ok351)* single-mutant animals and *cdk-8(tm1238)* single-mutant animals showed 0% (n >100) and 5% Eiv phenotype (n = 78), respectively.

An interesting observation was that the Eiv phenotype was such that the intestine, not the egg-laying apparatus, was spilled through the exploded vulva. One explanation for this phenotype could be that the cell cycles in the hypodermis or vulval cells were deregulated by *rnt-1* and *cdk-8* defects. To test this possibility, we examined the numbers of vulval precursor cells (VPCs) and of hypodermal seam cells. However, we could not identify significant differences (data not shown), suggesting that there might be other mechanisms that are regulated by both *rnt-1* and the CDK8 mediator complex near VPCs and hypodermal seam cells. It would be of interest to examine whether RNT-1 and the CDK8-containing mediator complex are involved in the regulation of signal transduction pathways such as TGFβ and WNT pathways, which may be responsible for the differentiation of hypodermal cells.

### *acs-4* is a putative target gene of RNT-1 and CDK8 mediator complex

**Figure 3:**
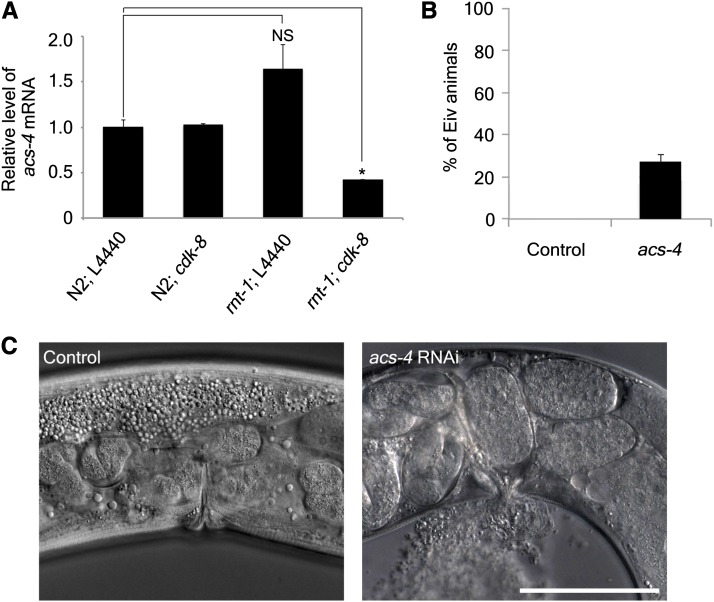
*acs-4* is a putative target gene of *rnt-1* and CDK8 mediator complex. (A) Relative mRNA level of *acs-4* detected by quantitative real-time PCR in the mixed stage of wild-type and *rnt-1* mutant animals with or without *cdk-8* RNAi. It was triplicated and normalized by the *act-1* gene. *p value <0.05. NS, not significant. (B) The percentage of exploded intestine through vulva (Eiv) animals when *acs-4* was knocked down by RNAi. (C) DIC images of the vulva region of the animals in which *acs-4* was knocked down by RNAi. White bar indicate a scale bar (50 μm).

To identify genes that are regulated by RNT-1 and the CDK8 mediator complex, we searched for genes that are reported to cause similar phenotype to our exploded intestine through the vulva phenotype. The keywords to extract the candidate genes from the WormBase were "exploded through vulva." Among them, we selected those with the *rnt-1* binding site(s) in their promoter regions (Supporting Information, Table S1). We then performed RNAi experiments of these candidate genes to determine whether their RNAi caused the same phenotype as *cdk-8* RNAi in *rnt-1* mutants. We also measured the amount of transcripts of putative target genes by real-time quantitative PCR to determine whether their transcription was dependent on RNT-1 and CDK8. We found that *acs-4* was a gene from the list that showed the Eiv phenotype and was coregulated by *rnt-1* and *cdk-8* (Figure 3A). The transcript of *acs-4* was decreased only when both *rnt-1* and *cdk-8* were knocked down. We confirmed that the Eiv phenotype was induced by RNAi of *acs-4* alone in the wild-type background (Figure 3, B and C) at a lower frequency than the *cdk-8* RNAi in the *rnt-1* mutant background, suggesting that *acs-4* is not the only target gene. *acs-4* encodes a fatty acyl CoA synthase and its role has been studied in terms of metabolism. Metabolic problems cause a variety of defects in development. Deprivation of fatty acids as a result of mutation in lipid metabolism causes developmental defects such as embryonic lethality and larval arrest in *C. elegans*, which are triggered by incomplete cytoskeletal components, reduction of function in signaling pathway, and abnormal biophysical property of membrane (Vrablik and Watts 2012). It has been recently reported that fatty-acid biosynthesis affects the polarity of intestine cells in *C. elegans* (Zhang *et al.* 2011). The intestine of *C. elegans* stores lots of fatty acids. It is conceivable that deregulation of *acs-4*, which is regulated by *rnt-1* and CDK8 mediator complex, may have led to abnormal lipid status of the worms, which has resulted in the Eiv phenotype in general.

In summary, our results suggest that *rnt-1* may physically interact with the CDK8 mediator complex in *C. elegans*. Consistent with our results, physical and genetic interactions between Lozenge, a RUNX gene, and CDK8 mediator complex have recently been reported in flies (Gobert *et al.* 2010). Taken together, we propose that the action mechanism of RUNX family proteins may be evolutionarily conserved, and that it would be of a great interest to establish the role of CDK8 mediator complex in the function of RUNX proteins in mammals.

## Supplementary Material

Supporting Information

## References

[bib1] Aberg T. Cavender A. Gaikwad J. S. Bronckers A. L. Wang X., 2004 Phenotypic changes in dentition of Runx2 homozygote-null mutant mice. J. Histochem. Cytochem. 52: 131–139 14688224 10.1177/002215540405200113

[bib2] Bae S. C. Lee Y. H., 2006 Phosphorylation, acetylation and ubiquitination: the molecular basis of RUNX regulation. Gene 366: 58–66 16325352 10.1016/j.gene.2005.10.017

[bib3] Banerjee C. McCabe L. R. Choi J. Y. Hiebert S. W. Stein J. L., 1997 Runt homology domain proteins in osteoblast differentiation: AML3/CBFA1 is a major component of a bone-specific complex. J. Cell. Biochem. 66: 1–8 9215522 10.1002/(sici)1097-4644(19970701)66:1<1::aid-jcb1>3.0.co;2-v

[bib4] Bangsow C. Rubins N. Glusman G. Bernstein Y. Negreanu V., 2001 The RUNX3 gene–sequence, structure and regulated expression. Gene 279: 221–232 11733147 10.1016/s0378-1119(01)00760-0

[bib5] Blyth K. Cameron E. R. Neil J. C., 2005 The RUNX genes: gain or loss of function in cancer. Nat. Rev. Cancer 5: 376–387 15864279 10.1038/nrc1607

[bib6] Braun T. Woollard A., 2009 RUNX factors in development: lessons from invertebrate model systems. Blood Cells Mol. Dis. 43: 43–48 19447650 10.1016/j.bcmd.2009.05.001

[bib7] Brenner S., 1974 The genetics of Caenorhabditis elegans. Genetics 77: 71–94 4366476 10.1093/genetics/77.1.71 PMC1213120

[bib8] Casamassimi A. Napoli C., 2007 Mediator complexes and eukaryotic transcription regulation: an overview. Biochimie 89: 1439–1446 17870225 10.1016/j.biochi.2007.08.002

[bib9] Coffman J. A., 2003 Runx transcription factors and the developmental balance between cell proliferation and differentiation. Cell Biol. Int. 27: 315–324 12788047 10.1016/s1065-6995(03)00018-0

[bib10] Cohen M. M. Jr, 2009 Perspectives on RUNX genes: an update. Am. J. Med. Genet. A. 149A: 2629–2646 19830829 10.1002/ajmg.a.33021

[bib11] Fukamachi H. Ito K., 2004 Growth regulation of gastric epithelial cells by Runx3. Oncogene 23: 4330–4335 15156189 10.1038/sj.onc.1207121

[bib12] Gobert V. Osman D. Bras S. Auge B. Boube M., 2010 A genome-wide RNA interference screen identifies a differential role of the mediator CDK8 module subunits for GATA/ RUNX-activated transcription in Drosophila. Mol. Cell. Biol. 30: 2837–2848 20368357 10.1128/MCB.01625-09 PMC2876525

[bib13] Kagoshima H. Shigesada K. Kohara Y., 2007 RUNX regulates stem cell proliferation and differentiation: insights from studies of C. elegans. J. Cell. Biochem. 100: 1119–1130 17265434 10.1002/jcb.21174

[bib14] Kahn N. W. Rea S. L. Moyle S. Kell A. Johnson T. E., 2008 Proteasomal dysfunction activates the transcription factor SKN-1 and produces a selective oxidative-stress response in Caenorhabditis elegans. Biochem. J. 409: 205–213 17714076 10.1042/BJ20070521

[bib15] Lee J. Ahnn J. Bae S. C., 2004 Homologs of RUNX and CBF beta/PEBP2 beta in C. elegans. Oncogene 23: 4346–4352 15156192 10.1038/sj.onc.1207669

[bib16] Lee K. Shim J. Bae J. Kim Y. J. Lee J., 2012 Stabilization of RNT-1 protein, runt-related transcription factor (RUNX) protein homolog of Caenorhabditis elegans, by oxidative stress through mitogen-activated protein kinase pathway. J. Biol. Chem. 287: 10444–10452 22308034 10.1074/jbc.M111.314146 PMC3323012

[bib17] Leroux M. R. Candido E. P., 1995 Characterization of four new tcp-1-related cct genes from the nematode Caenorhabditis elegans. DNA Cell Biol. 14: 951–960 7576182 10.1089/dna.1995.14.951

[bib18] Lincke C. R. The I. van Groenigen M. Borst P., 1992 The P-glycoprotein gene family of Caenorhabditis elegans. Cloning and characterization of genomic and complementary DNA sequences. J. Mol. Biol. 228: 701–711 1360540 10.1016/0022-2836(92)90855-e

[bib19] Michaud J. Wu F. Osato M. Cottles G. M. Yanagida M., 2002 In vitro analyses of known and novel RUNX1/AML1 mutations in dominant familial platelet disorder with predisposition to acute myelogenous leukemia: implications for mechanisms of pathogenesis. Blood 99: 1364–1372 11830488 10.1182/blood.v99.4.1364

[bib20] Mundlos S. Otto F. Mundlos C. Mulliken J. B. Aylsworth A. S., 1997 Mutations involving the transcription factor CBFA1 cause cleidocranial dysplasia. Cell 89: 773–779 9182765 10.1016/s0092-8674(00)80260-3

[bib21] Nam S. Jin Y. H. Li Q. L. Lee K. Y. Jeong G. B., 2002 Expression pattern, regulation, and biological role of runt domain transcription factor, run, in Caenorhabditis elegans. Mol. Cell. Biol. 22: 547–554 11756550 10.1128/MCB.22.2.547-554.2002 PMC139740

[bib22] Nimmo R. Antebi A. Woollard A., 2005 mab-2 encodes RNT-1, a C. elegans Runx homologue essential for controlling cell proliferation in a stem cell-like developmental lineage. Development 132: 5043–5054 16236764 10.1242/dev.02102

[bib23] Osato M., 2004 Point mutations in the RUNX1/AML1 gene: another actor in RUNX leukemia. Oncogene 23: 4284–4296 15156185 10.1038/sj.onc.1207779

[bib24] Osato M. Asou N. Abdalla E. Hoshino K. Yamasaki H., 1999 Biallelic and heterozygous point mutations in the runt domain of the AML1/PEBP2alphaB gene associated with myeloblastic leukemias. Blood 93: 1817–1824 10068652

[bib25] Otto F. Thornell A. P. Crompton T. Denzel A. Gilmour K. C., 1997 Cbfa1, a candidate gene for cleidocranial dysplasia syndrome, is essential for osteoblast differentiation and bone development. Cell 89: 765–771 9182764 10.1016/s0092-8674(00)80259-7

[bib26] Schroeder L. K. Kremer S. Kramer M. J. Currie E. Kwan E., 2007 Function of the Caenorhabditis elegans ABC transporter PGP-2 in the biogenesis of a lysosome-related fat storage organelle. Mol. Biol. Cell 18: 995–1008 17202409 10.1091/mbc.E06-08-0685 PMC1805080

[bib27] Shim J. Lee J., 2008 Regulation of rnt-1 expression mediated by the opposing effects of BRO-1 and DBL-1 in the nematode Caenorhabditis elegans. Biochem. Biophys. Res. Commun. 367: 130–136 18158917 10.1016/j.bbrc.2007.12.097

[bib28] Song W. J. Sullivan M. G. Legare R. D. Hutchings S. Tan X., 1999 Haploinsufficiency of CBFA2 causes familial thrombocytopenia with propensity to develop acute myelogenous leukaemia. Nat. Genet. 23: 166–175 10508512 10.1038/13793

[bib29] Thastrup O. Cullen P. J. Drobak B. K. Hanley M. R. Dawson A. P., 1990 Thapsigargin, a tumor promoter, discharges intracellular Ca2+ stores by specific inhibition of the endoplasmic reticulum Ca2(+)-ATPase. Proc. Natl. Acad. Sci. USA 87: 2466–2470 2138778 10.1073/pnas.87.7.2466 PMC53710

[bib30] Vrablik T. L. Watts J. L., 2012 Emerging roles for specific fatty acids in developmental processes. Genes Dev. 26: 631–637 22474257 10.1101/gad.190777.112 PMC3323873

[bib31] Wang Q. Stacy T. Binder M. Marin-Padilla M. Sharpe A. H., 1996 Disruption of the Cbfa2 gene causes necrosis and hemorrhaging in the central nervous system and blocks definitive hematopoiesis. Proc. Natl. Acad. Sci. USA 93: 3444–3449 8622955 10.1073/pnas.93.8.3444 PMC39628

[bib32] Werner M. H. Shigesada K. Ito Y., 1999 Runt domains take the lead in hematopoiesis and osteogenesis. Nat. Med. 5: 1356–1357 10581073 10.1038/70920

[bib33] Zhang H. Abraham N. Khan L. A. Hall D. H. Fleming J. T., 2011 Apicobasal domain identities of expanding tubular membranes depend on glycosphingolipid biosynthesis. Nat. Cell Biol. 13: 1189–1201 21926990 10.1038/ncb2328 PMC3249144

