# Supplementary material for: Identification of Genes Interacting with rnt-1 Through Large-Scale RNAi Screening in Caenorhabditis elegans
Source: G3 (Bethesda). 2013 Oct 1;3(10):1779–84. doi: 10.1534/g3.113.007898 (PMC3789802; doi:10.1534/g3.113.007898)
Supplement: Supporting Information [file supp_3_10_1779__index.html]

Identification of Genes Interacting with rnt-1 Through Large-Scale RNAi Screening in Caenorhabditis elegans — Supporting Information 

# Identification of Genes Interacting with *rnt-1* Through Large-Scale RNAi Screening in *Caenorhabditis elegans*

## Supporting Information for Lee *et al.*, 2013

**Files in this Data Supplement:**

- Table S1 - The list of genes that were selected with the keyword of 'exploded through vulva' and have putative *rnt-1* binding sequences in their promoter regions (PDF, 486 KB)
