# Supplementary material for: Identification of Genes Interacting with rnt-1 Through Large-Scale RNAi Screening in Caenorhabditis elegans
Source: G3 (Bethesda). 2013 Oct 1;3(10):1779–84. doi: 10.1534/g3.113.007898 (PMC3789802; doi:10.1534/g3.113.007898)
Supplement: Supporting Information [file supp_g3.113.007898_007898SI.pdf]

G3·Genes | Genomes | Genetics

Identification of genes interacting with *rnt-1* through large-scale RNAi screening in *Caenorhabditis elegans*

Kiho Lee[*, 1, 3], Jiwon Shim[*, 1, 4], Jihyun Lee[*, 1] Junho Lee[*, §, 2]

[*]Department of Biological Sciences, **[§]**Department of Biophysics and Chemical Biology, Seoul National University, Seoul, South Korea 151-742
[1]These authors equally contributed to this work.
[2] Corresponding author
[3] Department of Medicine, Infectious Diseases Division, Rhode Island Hospital, Warren Alpert Medical School of Brown University, 593 Eddy Street, Providence, RI 02903, USA
[4] Department of Molecular, Cell and Developmental Biology, University of California, Los Angeles, California 90095, USA

DOI: 10.1534/g3.113.007898

Table S1 The list of genes that were selected with the keyword of 'exploded through vulva' and have putative *rnt-1* binding sequences in their promoter regions.

Gene Name	RNT-1 binding sites from ATG	Description
spd-1	-1695	Protein regulator of cytokinesis 1 isoform 3
mek-2	-469, -599	Dual specificity mitogen-activated protein kinase kinase 2
cogc-1	-538	Conserved oligomeric Golgi complex subunit 1
Y47G6A.9	-282, -798	DNA-directed RNA polymerase III subunit RPC7
inx-12	-1362	Innexin
R12E2.2	-214	Chromosome 1 open reading frame 9, isoform CRA_b
Y110A7A.8	-1266, -1932	Isoform 1 of U4/U6 small nuclear ribonucleoprotein Prp31
F48C1.4	-526	
T08B2.8	-286, -1495	39S ribosomal protein L23, mitochondrial
san-1	-1352	Mitotic checkpoint serine/threonine-protein kinase BUB1
rab-10	-1033	Ras-related protein Rab-10
unc-37	-1541, -1634	Transducin-like enhancer protein 1
teg-4	-198	Isoform 1 of Splicing factor 3B subunit 3
H27M09.1	-235	DEAD-box protein abstrakt variant (Fragment)
C30F12.1	-11	Ribonuclease Zc3h12a-like
unc-120	-1716	Serum response factor
hsr-9	-183	Isoform 1 of Tumor suppressor p53-binding protein 1
ned-8	-1027	NEDD8
ubc-25	-726, -1812	Isoform 1 of Ubiquitin-conjugating enzyme E2 Q2
wve-1	-1656	Wiskott-Aldrich syndrome protein family member 3
F33H2.5	-374, -505, -1697	DNA polymerase epsilon catalytic subunit A
F09D1.1	-928	U4/U6.U5 tri-snRNP-associated protein 2
Y49F6B.2	-429	Deoxynucleotidyltransferase terminal-interacting protein 2
cyh-1	-616	Cyclin-H
fkh-6	-831	Isoform 1 of Forkhead box protein I1
dsh-2	-245, -1811	Segment polarity protein dishevelled homolog DVL-2
dmd-5	-1411	Doublesex- and mab-3-related transcription factor A1
fbf-2	-471, -1185	Isoform 1 of Pumilio homolog 1
wrn-1	-572	Werner syndrome ATP-dependent helicase
cul-4	-1091, -1284, -1713	Isoform 2 of Cullin-4A
F37B12.1	-1453	
pax-3	-1761	Isoform 3 of Paired box protein Pax-4
Y19D2B.1	-908	Tubulin alpha-1C chain
npp-3	-514, -640	Nuclear pore complex protein Nup205
arp-1	-1650	Alpha-centractin
Y53G8AR.9	-1112, -1203	Zinc finger CCCH domain-containing protein 10
cri-3	-1725	Complement component 1 Q subcomponent-binding protein,
T02C12.2	-1926	snRNA-activating protein complex subunit 3
rnr-2	-1297	Isoform 1 of Ribonucleoside-diphosphate reductase subunit
R144.11	-644	
paa-1	-648	Serine/threonine-protein phosphatase 2A 65 kDa regulatory
abi-1	-1368	Isoform 1 of Abl interactor 1
dlc-1	-1833	Dynein light chain 2, cytoplasmic
let-765	-734	Strawberry notch homolog 1
isw-1	-955, -1555	Isoform 2 of Probable global transcription activator SNF2L1
acs-4	-1499	Isoform Long of Long-chain-fatty-acid--CoA ligase 4
mdt-30	-1316	Replication factor C subunit 5 isoform 3
sas-4	-105, -459	Isoform 3 of Filamin-A-interacting protein 1
R08D7.1	-484	Isoform 2 of BUD13 homolog
cls-2	-1641	Isoform 1 of CLIP-associating protein 1
ina-1	-1604	Isoform Alpha-6X1A of Integrin alpha-6
ZK1128.3	-520, -729	
D2045.9	-697	Procollagen galactosyltransferase 2
col-92	-1269, -1619, -1802	Collagen alpha-1(I) chain
pqn-84	-334	Isoform 7 of Dermokine
M57.2	-818	Geranylgeranyl transferase type-2 subunit alpha
arp-11	-62	
D2096.8	-1655	Nucleosome assembly protein 1-like 1
D1046.2	-1134	
dcaf-1	-1098	Isoform 1 of Protein VPRBP

mtr-4	-1082	Superkiller viralicidic activity 2-like 2
T11G6.8	-459	Isoform 1 of Pre-mRNA-splicing factor RBM22
mep-1	-771	Zinc finger, C2H2-type
ent-1	-1675	Isoform 1 of Equilibrative nucleoside transporter 3
col-133	-397	Collagen alpha-1(I) chain
sfa-1	-917	Isoform 1 of Splicing factor 1
ZK550.4	-1166	General transcription factor IIE subunit 1
dnj-22	-431, -1202	DnaJ homolog subfamily C member 17
ehbp-1	-770	Isoform 2 of EH domain-binding protein 1
cash-1	-1646	Isoform Beta of Striatin-3
syx-5	-179, -596, -629, -1057	Isoform 1 of Syntaxin-5
F43D2.1	-518	Isoform 1 of Cyclin-K
prmt-1	-270	protein arginine N-methyltransferase 1 isoform 1
sft-4	-1358	Surfeit 4
dpy-22	-1980	Mediator of RNA polymerase II transcription subunit 12
F41E7.1	-1055	Isoform 1 of Sodium/hydrogen exchanger-like domain-
ain-1	-1008, -1920	Isoform 7 of Dermokine
osm-11	-372, -869	
hlh-28	-310, -1362	Helix-loop-helix DNA-binding domain, bHLH)
